# Utilizing the Health Belief Model to understand heat mitigation behaviors in the United States: Results of an online panel survey

**DOI:** 10.1371/journal.pone.0334697

**Published:** 2025-10-14

**Authors:** Bibiana Martinez, Celeste Beck, Jo Kay Ghosh

**Affiliations:** Department of Research and Evaluation, Heluna Health, City of Industry, California, United States of America; Hamadan University of Medical Sciences, IRAN, ISLAMIC REPUBLIC OF

## Abstract

The factors impacting individual-level heat mitigation behaviors (e.g., seeking shade, staying cool, wearing loose-fitting clothes) during extreme heat events among adults in the United States are poorly understood. The Health Belief Model (HBM) has been used extensively to explore health promoting behaviors; we explored the application of the HBM constructs to understand heat mitigation behaviors among U.S. adults. Online panel data from the Household Emergency Preparedness Survey was collected in May 2024 to explore knowledge, attitudes, beliefs and behaviors related to extreme heat mitigation among a nationally representative sample of U.S. adults. An outcome variable assessing likeliness to engage in heat mitigation behaviors was developed by dichotomizing a sum of responses to questions asking about likelihood to engage in different mitigation behaviors; scores were split on the median value to classify those “more likely” versus “less likely” to engage in mitigation behaviors. Descriptive statistics were used to explore HBM construct responses among our sample; logistic regression models examined whether perceived benefits, perceived barriers, perceived susceptibility, perceived severity, self-efficacy, and cues to action were associated with more likelihood to engage in heat mitigation behaviors, after controlling for age, sex, income, race/ethnicity, education, and political affiliation. We included 6095 responses to the online panel survey in our analysis. A multivariable logistic regression model including all HBM constructs and adjusting for sociodemographic characteristics found that higher degrees of perceived benefits (OR=1.30, 95% CI:1.22–1.39), self-efficacy (OR=3.69, 95% CI: 3.26–4.10), and cues to action (OR=1.48, 95% CI:1.38–1.60) were positively associated with being more likely to engage in heat mitigation behaviors. Results from our analyses suggest that communication strategies, guidelines, and interventions which incorporate cues to action, as well as those focused on improving perceived benefits and self-efficacy can be most effective in improving heat mitigation behaviors.

## Introduction

Extreme heat events are becoming more frequent as a result of climate change [1,2]. While there is no standardized definition for what constitutes an extreme heat event, they generally refer to periods of sustained and uncomfortable hot weather, with temperatures higher than normal for that area and season, lasting between 1–5 days [1,3]. Heat has been shown to impact human health in a variety of ways; it is the primary cause of weather-related mortality and has been linked with increases in morbidity from chronic conditions, pregnancy, and mental health [1,4–7]. Vulnerable populations, including older adults, children, individuals with multiple comorbidities, pregnant individuals, and individuals with low socioeconomic status are at increased risk for these impacts [1,8–10]. During the past two decades, the number of heat-related deaths among individuals older than 65 years has increased by approximately 85% [7,11]. As the number of people exposed and impacted by extreme heat continues to grow, the need to prevent and mitigate its health impacts has become urgent [7].

While our understanding of the impact of extreme heat on health is growing, less is known about the drivers of individual-level mitigation behaviors for extreme heat events. Public health agencies, including the Centers for Disease Control and Prevention and the World Health Organization, provide behavioral recommendations on how to mitigate the impact of exposure to extreme heat [7,12]. In particular, recommended mitigation actions include: staying cool and/or keeping your home cool (by running an air conditioner, staying in an air-conditioned location, covering windows with curtains or blinds, or going to a cooling center); keeping the body cool and hydrated (by drinking plenty of fluids and/or wearing loose-fitting lightweight clothes); and staying out of the heat (seeking shade when outside, avoiding strenuous outdoor activities, and staying indoors during the hottest part of the day).

The Health Belief Model (HBM) is a framework first conceived in the 1950’s by social psychologists in the United States Public Health Service to help explain health prevention behaviors [13]. Since then, it has been widely used to understand the factors that impact health behaviors [14–18]. The HBM posits that key beliefs about health conditions and behaviors can predict individuals’ health-related behaviors. These key constructs include individuals’ perceived benefits, perceived barriers, perceived severity, and perceived susceptibility (sometimes combined into perceived threat), as well as an individual’s assessment of their self-efficacy, and cues to action (specific events/behaviors which trigger a behavior) which together are hypothesized to lead to a specific health-related action.

Research exploring climate change mitigation behaviors worldwide has found associations between some constructs of the Health Belief Model and mitigation behaviors. In particular, perceived benefits, cues to action, self-efficacy and perceived severity have been found to impact mitigation behaviors [19–22]. Similarly, certain sociodemographic factors have been linked to mitigation behaviors, especially sex, income, political affiliation, age, and education [10,23–25]. Furthermore, a study of older adults in New York City found that both risk perceptions and perceived barriers impacted their air conditioning use [26]. However, little other research has explored the drivers of individual-level extreme heat mitigation behaviors in the United States, especially during the last decade.

This study aims to explore the utility of the Health Belief Model constructs in explaining mitigation behaviors for extreme heat among adults in the United States. We hypothesized that mitigation behaviors would be positively associated with perceived benefits of those behaviors, perceived susceptibility and severity of the impact of extreme heat, self-efficacy, and cues to action about extreme heat behaviors, while being negatively associated with perceived barriers to extreme heat mitigation behaviors.

## Materials and methods

### Study sample

We conducted a cross-sectional study using data from the Household Emergency Preparedness Survey, an online panel survey administered to U.S. adults by Heluna Health from May 17 through June 11, 2024, which assessed attitudes and likelihood of mitigation behaviors related to extreme heat events. Survey respondents were recruited using an opt-in web panel service from Dynata, LLC, which incentivizes panel participation using a point-based system. To achieve a nationally representative sample of U.S. adults, survey responses were census-balanced and calibrated to a general adult population. Post stratification weights were used to reduce sampling and non-response bias with respect to the sociodemographic characteristics of the U.S. population. We oversampled some sociodemographic groups to account for lower response rates, as identified in a review of some online surveys [27]. These methods were used in previous iterations of the survey, and have been described in detail elsewhere [28]. This survey was found to be exempt from full Institutional Review Board (IRB) review by the Salus IRB (45 CFR 46.104(d), Category 2: Educational Tests, Surveys, Interviews & Observation). We used the following informed consent process: prior to commencing the survey, respondents were presented with a brief summary of the study objectives, as well as risks and benefits of participation, and were asked to click a button to proceed which indicated consent to participate in the study.

We collected 6223 responses to the online survey from adult individuals residing in the U.S. Given that many of the mitigation behaviors explored do not apply to unhoused individuals, defined as those living in the street, in a car, or in a shelter (e.g., operating an AC system, covering windows with curtains, etc.), records for individuals who reported those housing conditions (N = 24, 0.4% of all respondents) and for those who did not provide information about their housing status (N = 104, 1.7% of all respondents) were excluded from our analytic sample. Our final analytic sample included 6095 respondents (98% of all survey participants).

### Measures

#### Likelihood of extreme heat mitigation behaviors.

To create a summary score of heat mitigation behaviors, we summed responses to 8 questions asking participants to rate their likelihood to take recommended actions (see Table 1) if impacted by an extreme heat event. Responses for each question were provided on a Likert scale from 1–5 (1 = Not at all likely to take action, 5 = Extremely likely to take action) (Cronbach’s alpha = 0.88). In line with Akompab and colleagues [19], this summary score was dichotomized using the median value as a threshold to create low (“Less likely to engage in mitigation behaviors”) and high (“More likely to engage in mitigation behaviors”) categories; this binary variable was used as our analytic outcome.

**Table 1:** Health Belief Model constructs and questions.

Heat Mitigation Behaviors	Imagine that extreme heat (abnormally high heat and/or humidity lasting at least 2–3 days) were affecting you where you live now. How likely would you be to take these specific actions?
	Run air conditioning in my home to help control the temperature
	Cover windows with curtains or blinds
	Go to a cooling center or other air-conditioned building during the day
	Drink a lot of fluids to stay hydrated
	Avoid strenuous outdoor exercise
	Avoid going outside during the hottest part of the day
	Stay in the shade when outside
	Wear light, loose-fitting clothes
Perceived Benefits	Imagine that extreme heat is happening where you live now. Do you believe the following actions would be beneficial to you during the heat?
	Running air conditioning in my home to help control the temperature
	Covering windows with curtains or blinds
	Drinking a lot of fluids to stay hydrated
	Avoiding strenuous outdoor exercise
	Going to a cooling center or to a building in my community that has air conditioning
	Avoiding going outside during the hottest time of the day
	Staying in the shade when outside
	Wearing light, loose-fitting clothes
Perceived Barriers	Please rate your level of agreement with the following statements, as they relate to barriers or challenges to being more prepared for extreme heat.
	I don’t have enough money to prepare.
	I don’t have personal transportation.
	I can’t make necessary modifications to my home (drafts, air conditioning).
	I don’t open my windows at night to allow air to enter during extreme heat (due to security reasons or air pollution)
Perceived Severity	Imagine that extreme heat is happening where you live now. Please rate your agreement/disagreement with the following statements:
	I may become ill if my body temperature becomes elevated due to the heat.
	If my body temperature rises rapidly and I am unable to cool down, I may need to go to the emergency room.
	If I experience dehydration due to the heat, this may cause long-term damage to my health.
	I may be exposed to higher concentrations of air pollutants due to the heat.
	I may become anxious, depressed, or distressed due to the heat.
Perceived Susceptibility	How likely do you think that extreme heat is to happen in the area that you live within the next 12 months?
	If extreme heat does occur, how likely do you think that extreme heat is to personally impact you within the next 12 months?
Self-Efficacy	People may find taking action in response to extreme heat difficult because they may not have the money, resources, or tools required to take action. On a scale of 1–5, please indicate whether you are able to take these actions during extreme heat.
	Run air conditioning in my home to help control the temperature
	Cover windows with curtains or blinds
	Go to a cooling center or other air-conditioned building during the day
	Drink a lot of fluids to stay hydrated
	Avoid strenuous outdoor exercise
	Avoid going outside during the hottest part of the day
	Stay in the shade when outside
	Wear light, loose-fitting clothes
Cues to Action	Please rate your agreement with the following statements, as they pertain to actions that you would take in the following situations relating to extreme heat.
	If I see in a weather forecast that it is going to be a very hot day, I try to stay in cool environments.
	Seeing someone taken to the hospital for heat-related illness on the news would motivate me to protect myself on very hot days.
	If my doctor advised me to avoid outdoor exercise on very hot days, I would listen.
	If I saw friends taking actions to protect themselves during extreme heat, that would motivate me to take protective actions also.
	If I received a weather alert on my phone, radio, or TV, it would motivate me to take protective actions during extreme heat.
	If a friend/neighbor/family member reached out to express concern about my wellbeing due to heat, I would take protective actions.

#### Perceived benefits.

The online survey asked respondents whether they believed the specific 8 actions listed in the previous section (see Table 1) would be beneficial to them if extreme heat were happening where they live. Answer choices included a 1–5 Likert response scale (1 = Not beneficial and 5 = A very big benefit). Answers were summed to create a single variable ranging from 8–40, with higher scores representing more perceived benefits of heat mitigation behaviors (Cronbach’s alpha = 0.88).

#### Perceived barriers.

We asked survey respondents a set of questions related to preparedness and response to extreme heat events. Even though the question stem is worded to inquire about barriers to preparedness, the items mentioned in the questions align with factors commonly cited in the literature as barriers to mitigation behaviors (see Table 1) [19,20,26,29]. Response options for all questions included a 1–5 Likert response scale (1 = Strongly disagree and 5 = Strongly agree). Responses to these questions were summed to create a variable with values ranging from 5–25, with higher scores denoting more barriers (Cronbach’s alpha = 0.75).

#### Perceived susceptibility.

To assess perceived susceptibility, we included two questions asking about likelihood of increased frequency and personal impact of extreme heat events in the next 12 months (Table 1). For both questions, response options were 1–5 on a Likert scale, (1 = No likelihood of happening/impacting, 5 = Very high likelihood of happening/impacting). A variable was created using the product of these two questions as our assessment of perceived susceptibility (range 1–25, Cronbach’s alpha = 0.81).

#### Perceived severity.

Perceived severity was assessed by summing responses to questions asking about participant agreement with statements about the mental and physical impact of extreme heat on them (see Table 1). A Likert scale (1–5, 1 = Strongly disagree, 5 = Strongly agree) provided response choices for these questions. Answers were summed into a single variable representing perceived severity (range 5–25, Cronbach’s alpha = 0.86).

#### Self-efficacy.

We assessed self-efficacy by asking participants to state whether they were able to conduct the mitigation behaviors included in our outcome variable (see Table 1). Response choices included a 1–5 Likert scale with answers ranging from ‘Not able to take action’ (1), to ‘Extremely able to take action’ (5). Responses were summed to create a self-efficacy variable with answers ranging from 8 to 40, with higher scores denoting more self-efficacy (Cronbach’s alpha = 0.90).

#### Cues to action.

We created a variable to represent the cues to action construct by summing participant responses to questions inquiring about participant agreement as to whether different prompts would lead them to conduct heat mitigation behaviors (see Table 1, Cronbach’s alpha = 0.89). Answers ranged from 6–30, with higher scores indicating more responsiveness to cues to action.

#### Demographic covariates.

Our analyses controlled for demographic variables previously reported to impact vulnerability, awareness, or perceptions of extreme heat [10,30], or engagement in extreme heat mitigation behaviors [19,23]. These included sex at birth (male vs. female); age (answer choices included ages 18–29, 30–39, 40–49, 50–64, and 65 and older); household income (answer choices: ≤ $34,999, $35,000- $54,999, $55,000-$99,999, ≥ $100,000), and education (less than a high school diploma; high school diploma/GED; some college/2 year degree; 4 year degree; graduate degree). We also included a variable representing political affiliation, with three categories: Democrat (including the response choices: strong Democrat; Democrat; and lean Democrat); Independent; and Republican (including the response choices: lean Republican; Republican; and strong Republican).

Additionally, our analyses controlled for race/ethnicity using a binary variable, categorizing respondents either as non-Hispanic White (NHW) or person of color (POC), which included individuals who identified as: Hispanics of all races, non-Hispanic Black/African-American, non-Hispanic Asian, non-Hispanic Middle Eastern/North African, non-Hispanic Native Hawaiian/Pacific Islander, non-Hispanic American Indian/Alaska Native, and 2 or more races. We opted for this binary categorization of race/ethnicity to reflect our understanding that the primary pathway through which race and ethnicity impact extreme heat exposure is through the experiences of racism and/or the legacy of structural racism (e.g., residence in neighborhoods with poor shade [31–33], lower rates of homeownership [34], etc.) [35].

### Statistical analysis

We used descriptive and summary statistics (frequencies, means, etc.) to characterize our study sample and explore the distribution of extreme heat mitigation behaviors, as well as the variables created to assess perceived barriers, benefits, susceptibility, severity, self-efficacy, and cues to action (predictors). All predictors were rescaled on a 0–10 scale to facilitate comparisons across constructs of analytic results. Bivariate logistic regression was used to explore associations between each HBM construct and our outcome measure of heat mitigation behaviors (more vs. less likely), as well as sociodemographic variables; multivariable logistic regression models explored the association between being more likely to engage in heat mitigation behaviors and all HBM constructs as well as our covariates of interest (alpha = 0.05). Since our outcome variable had no missing observations within our analytic sample, we conducted a complete-case analysis. We explored correlations among predictor variables in our multivariable models using a variance inflation factor (VIF) of 5 as a threshold for problematic correlation. To choose between multiple multivariable models, smaller Akaike information criterion (AIC) and larger R^2^ scores were used to determine better model goodness of fit. Odds ratios were back transformed to the original predictor scales in our results section for ease of interpretability. Survey weights were used in all our analyses, which were conducted using SAS/STAT software (SAS Institute Inc., Version 9.4.).

## Results

Approximately two-thirds of respondents identified as non-Hispanic White (60%), with the remaining sample identifying as a person of color (40%). Almost half of respondents were aged 50 or older (46%), more than half had household incomes above $55,000 (63%), and slightly more respondents were female (51%). Politically, 42% identified as Democrats, 33% as Republicans, and 26% as Independents. A detailed description of sample characteristics appears in Table 2.

**Table 2:** Sample sociodemographic characteristics and unadjusted logistic regression results modeling for more likeliness to engage in heat mitigation behaviors.

Characteristic	N (col %)^a^	Unadjusted OR* (95% CI**)
Age (years)		
18-29	1259 (20.4%)	0.44 (0.37-0.53)
30-39	1284 (17.5%)	0.58 (0.48-0.70)
40-49	1037 (15.9%)	0.63 (0.52-0.77)
50-64	1471 (24.0%)	0.77 (0.64-0.92)
65+	1077 (22.2%)	(REF)
Race/ethnicity		
Person of Color^b^	2767 (39.8%)	0.73 (0.65- 0.82)
Non-Hispanic White	3316 (59.9%)	(REF)
Missing	12 (0.3%)	
Sex		
Male	1892 (49.3%)	1.18 (1.05- 1.33)
Female	4203 (50.7%)	(REF)
Annual household income		
<$35,000	1934 (22.7%)	0.59 (0.51-0.69)
$35,000-$54,999	1206 (13.9%)	0.79 (0.67-0.94)
$55,000-$99,999	1644 (25.9%)	0.86 (0.74-1.01)
≥$100,000	1311 (37.5%)	(REF)
Political affiliation		
Democrat	2627 (41.9%)	1.05 (0.91-1.20)
Independent	1644 (25.6%)	0.79 (0.68-0.92)
Republican	1824 (32.5%)	(REF)
Education		
Less than high school diploma	140 (1.8%)	0.54 (0.36-0.81)
High school diploma/GED	1346 (18.1%)	0.70 (0.58-0.85)
Some college/2yr diploma	2092 (30.5%)	0.78 (0.65-0.93)
4yr college diploma	1601 (29.4%)	0.84 (0.69-1.00)
Graduate degree	916 (20.3%)	(REF)

*OR=Odds Ratio

**CI= 95% Confidence Interval

^a^N values were non-weighted; all other descriptive statistics were weighted.

^b^People of color category includes individuals identifying as: Hispanics of all races, non-Hispanic Black/African-American, non-Hispanic Asian, non-Hispanic Middle Eastern/North African, non-Hispanic Native Hawaiian/Pacific Islander, non-Hispanic American Indian/Alaska Native, and 2 or more races.

The likelihood of engaging in specific heat mitigation behaviors varied in our sample. Respondents were most likely to drink abundant fluids (mean = 4.43), to avoid strenuous outdoor activity (mean = 4.35), and to wear light, loose-fitting clothes (mean = 4.33), and were less likely to go to a cooling center (mean = 3.26) or run an air conditioning unit in their homes (mean = 4.18). Mean responses to each question appear on Table 3.

**Table 3:** Mean scores for heat mitigation behaviors.

*Imagine that extreme heat (abnormally high heat and/or humidity lasting at least 2–3 days) were affecting you where you live now. How likely would you be to take these specific actions?*	Mean (95% CI*)
Run air conditioning in my home to help control the temperature	4.18 (4.14-4.21)
Cover windows with curtains or blinds	4.21 (4.18-4.24)
Go to a cooling center or other air-conditioned building during the day	3.26 (3.22-3.30)
Drink a lot of fluids to stay hydrated	4.43 (4.40-4.45)
Avoid strenuous outdoor exercise	4.35 (4.33-4.38)
Avoid going outside during the hottest part of the day	4.31 (4.29-4.34)
Stay in the shade when outside	4.24 (4.22-4.27)
Wear light, loose-fitting clothes	4.33 (4.31-4.36)

*CI=Confidence Interval

The distribution of heat mitigation behavior categories (more vs. less likely) of our sample and their mean HBM construct scores by heat mitigation category are listed on Table 4. Slightly more than half of our sample (50.9%) were more likely to engage in mitigation behaviors. Except for the Perceived Barriers construct, we observed that mean scores were higher for respondents with more likeliness to engage in heat mitigation behaviors and lower for those categorized as being less likely to engage in heat mitigation behaviors. Among those more likely to engage in mitigation behaviors, the HBM constructs with highest scores in our sample were Self-Efficacy, Cues to Action, and Perceived Benefits (9.18, 8.59, and 8.25, respectively).

**Table 4:** Mean Health Belief Model constructs by heat mitigation category (more vs. less likely), and unadjusted odds ratio modeling for more likely to engage in heat mitigation behaviors.

HBM Construct	More likely (N = 2985) Mean (SE)	Less likely (N = 3110) Mean (SE)	Unadjusted OR*^,a^ (95% CI**)
Perceived Benefits	8.25 (0.03)	6.47 (0.04)	2.00 (1.90-2.10)
Perceived Barriers	2.21 (0.05)	3.11(0.05)	0.76 (0.73-0.80)
Perceived Susceptibility	4.84 (0.06)	4.22 (0.06)	1.07 (1.05-1.09)
Perceived Severity	6.36 (0.05)	6.01 (0.04)	1.07 (1.04-1.10)
Self-Efficacy	9.18 (0.02)	6.81 (0.04)	4.44 (4.04-4.87)
Cues to Action	8.59 (0.03)	7.25 (0.03)	2.22 (2.08-2.37)

*OR=Odds Ratio

**CI= 95% Confidence Interval

^a^Odds ratios resulted from modeling that used scaled (0–10) versions of the HBM constructs.

In our bivariate logistic regression results, respondents with higher perceived barrier scores had lowed odds of being more likely to engage in mitigation behaviors (OR=0.87, 95% CI:0.86–0.89). Conversely, higher odds of being more likely to engage in heat mitigation behaviors were associated with higher scores for the perceived benefits (OR=2.00, 95% CI:1.90–2.10), perceived susceptibility (OR=1.07, 95% CI:1.05–1.09), perceived severity (OR=1.07, 95% CI:1.04–1.10), self-efficacy (OR=4.44, 95% CI:4.04–4.87), and cues to action (OR=2.22, 95% CI:2.08–2.37) construct scores (see Table 4).

In multivariable logistic regression models including all constructs of the Health Belief Model simultaneously (Model 1, Table 5), the bivariate associations between each construct and the outcome of interest were all somewhat attenuated. Despite this attenuation, three HBM constructs (perceived benefits, self-efficacy, cues to action) remained positively associated with being more likely to engage in mitigation behaviors (Table 5). Meanwhile, perceived barriers, perceived susceptibility, and perceived severity showed no associations with the odds of more likeliness of engaging in mitigation behaviors. When additionally controlling for race/ethnicity, sex, political affiliation, age, and education (Model 2), the associations observed for each of the HBM constructs were similar as in Model 1, with a one unit increase in perceived benefits and cues to action increasing the odds of being more likely to engage in heat mitigation behaviors by about 30% (OR=1.30, 95% CI:1.22–1.39) and 48% (OR= 1.48, 95% CI:1.38–1.60), respectively, and with a one unit increase in self-efficacy score associated with more than 3 times the odds of being more likely to engage in heat mitigation behaviors (OR=3.69, 95% CI: 3.32–4.10). Additional covariate adjustment for household income did not modify the results but worsened our model fit statistics.

**Table 5:** Adjusted logistic regression models (modeling for more likeliness of engaging in mitigation behaviors). Model 1 includes only the HBM constructs, and Model 2 additionally adjusts for race/ethnicity, sex, political affiliation, age, and education.

	Model 1 AIC = 4412.13 R2 = 0.65	Model 2 AIC = 4412.11 R2 = 0.65
	Odds ratio^a^ (95% CI**)	Odds ratio^a^ (95% CI**)
Perceived Benefits	1.31 (1.23-1.39)	1.30 (1.22-1.39)
Perceived Barriers	1.01 (0.97-1.05)	1.00 (0.96-1.04)
Perceived Susceptibility	1.00 (0.97-1.03)	1.00 (0.97-1.03)
Perceived Severity	0.96 (0.92-1.01)	0.96 (0.91-1.01)
Self-efficacy	3.64 (3.28-4.04)	3.69 (3.32-4.10)
Cues to Action	1.51 (1.39-1.62)	1.48 (1.38-1.60)

^a^Odds ratios resulted from modeling that used scaled (0–10) versions of the HBM constructs.

**CI = Confidence Interval

Converting these results to the original survey measurement scales, for every one level increase in the original Likert scale of perceived benefits, self-efficacy, and cues to action, odds of being more likely to engage in heat mitigation behaviors increases by 9%, 50%, and 18%, respectively.

We performed sensitivity analyses to confirm the robustness of our results by creating two alternative versions of our outcome variable: instead of dichotomizing the variable into more/less likely to engage in behaviors using the median value of our continuous sum of extreme heat mitigation behaviors (cut point = 34), we used the 25^th^ and 75^th^ percentiles as dichotomizing thresholds (cut points 30 and 38, respectively). While the multivariable models using the 75th percentile threshold in the outcome variable found additional associations between race/ethnicity and political affiliation with likeliness of engaging in heat mitigation behaviors (for people of color vs. non-Hispanic White individuals, OR=1.41, 95% CI:1.16–1.71; and for individuals identifying as democrat vs. republican, OR=0.63, 95% CI: 0.51–0.79), the magnitude and direction of the associations of our final model remained consistent (see Supplemental Materials).

## Discussion

To our knowledge, our study is the first to use the Health Belief Model to explain heat mitigation behaviors among a representative sample of U.S. adults. As hypothesized, we found that perceived benefits, self-efficacy, and cues to action were positively associated with more likeliness of engaging in heat mitigation behaviors, with self-efficacy scores being most strongly associated with being more likely to engage in mitigation behaviors. We found no associations between more likeliness to engage in mitigation behaviors and the other components of the HBM (perceived barriers, perceived susceptibility, and perceived severity). Respondents reported being more likely to engage in some heat mitigation behaviors such as drinking more fluids, limiting strenuous outdoor activities, and wearing loose-fitting clothes, but reported being less likely to engage in behaviors associated with air conditioning use (either going to a cooling center or using a personal AC unit).

Some of our results align with previous research findings on climate change mitigation behaviors. Cues to action had been previously associated with heat and other climate change mitigation behaviors, including research using structural equation modeling that found internal cues to action to be associated with higher levels of AC use in Canada, as well as studies using multivariable models which found that higher cues to action were positively associated with good mitigation behaviors during heat waves in Australia [19,20,22]. In addition, this result is consistent with findings from a randomized controlled trial of a phone-based automatic heat advisory warning system among vulnerable adults in Montreal, Canada, which found that individuals who received phone alerts warning them about an upcoming extreme heat event were more likely to engage in recommended behaviors; the phone alerts, which acted as the cue to action, also provided a list of recommended behaviors [22]. These as well as our findings suggest that cues to action such as weather forecasts and alerts, medical recommendations, and mitigation suggestions from friends and family can be impactful components of guidelines and interventions to improve heat mitigation behaviors among adults in the U.S. It highlights the importance of multi-sectoral collaborations in our collective response to extreme heat events, as some of these cues to action would require coordination across fields which do not frequently work together (i.e., meteorologists, medical providers, and community members).

Our findings regarding self-efficacy and the perceived benefits of mitigation actions align with previous findings related to their drivers, which found both of these HBM constructs to be important drivers of heat mitigation behaviors and behavioral intentions. Richard and colleagues found that perceived benefits of AC use were associated with level of AC use among non-institutionalized adults living with chronic diseases in Canada, and van Valkengoed’s research showed strong relationships between self-efficacy and intent to engage in climate change related mitigation behaviors in the Netherlands [20,21]. Similarly, Akombap’s research in Adelaide, Australia found that perceived benefits were positively associated with good adaptative behaviors during heat waves among an adult sample (ages 30–69) [19]. These associations are noteworthy, because a single education campaign could focus on improving perceptions of the benefits and effectiveness of specific actions and the ability of individuals to take these actions, which could lead to the improved mitigation behaviors that buffer the impacts of extreme heat events. A special focus on strategies to improve self-efficacy may be warranted, as it was the construct with the strongest association with more likeliness to engage in heat mitigation behaviors in our analyses.

Our study has some limitations which are important to note. Given our use of an online panel survey, the generalizability of our results may be limited to individuals who have internet access and those who participate in online surveys and panels, potentially introducing selection bias. However, previous studies employing similar online panels have been shown to closely approximate results derived from multi-modal studies using a combination of phone, mail, and online survey modes [27,36]. Our survey defined extreme heat as “abnormally high heat and/or humidity lasting at least 2-3 days”; the ambiguity of this definition and variation in local experiences of heat likely reduced our measurement precision. Our survey asked questions about behavioral intent only, which can be different from actual behavior [21]; we expect that responses may overestimate adherence and that there would likely be an attenuation of the effects of the HBM constructs if assessed against actual actions taken. Moreover, participants could have overreported their likelihood to engage in protective behaviors due to social desirability bias, which might have also attenuated the associations we observed. Furthermore, our survey did not ask direct questions about perceived barriers to heat mitigation behaviors, including practical barriers such as high electricity costs or lack of access to cooling centers, instead asking participants to rate their agreement with statements related to general preparedness for extreme heat. However, we believe these variables appropriately capture many of the barriers to mitigation behaviors described in the literature. Additionally, the Health Belief Model focuses on cognitive constructs, so our study does not address the potential emotional, social, and cultural factors which may drive heat mitigation behaviors. Lastly, given the cross-sectional nature of our research, our analyses cannot determine the causal relationships between Health Belief Model constructs and heat mitigation behaviors. Our study did not directly test or evaluate any specific heat prevention intervention, which limits the practical implications of our research; further studies should focus on evaluating actual heat prevention strategies to identify whether the use of HBM constructs (i.e., perceived benefits, self-efficacy, cues to action) in intervention design can improve uptake of heat prevention behaviors and attitudes. Future research should try to replicate our findings among different populations (such as those without internet access). Our understanding of the factors impacting heat mitigation behaviors would also benefit from longitudinal studies which explore the causal pathways of heat mitigation behaviors more in depth, such as assessing how behavioral intent aligns with the actions people have taken during extreme heat events. The impact of environmental, economic, and social factors on extreme heat mitigation behaviors should also be explored. Qualitative research can also be an important tool in deepening our understanding of the ways in which lived experiences impact such behaviors.

While heat related illness is highly preventable, it requires individuals to engage in specific behaviors to mitigate risks. Our online survey collected data from a representative sample of U.S. adults, allowing us to identify national attitudes and behavioral intent regarding extreme heat and prevention behaviors. By using the Health Belief Model to understand heat mitigation behaviors, we utilized a well-established health promotion framework to identify the specific drivers of health prevention behaviors which can be most effectively leveraged to develop effective, broad campaigns to improve awareness and knowledge about heat prevention behaviors among the general public.

Our results contribute important insights to the field of heat mitigation behavior, especially among adults in the general U.S. population. They suggest the potential effectiveness of strategies well within the arsenal of public health professionals (educational campaigns; behavioral nudges), and can provide guidance to policy makers, practitioners, and stakeholders in optimizing communication strategies, developing guidelines, and designing interventions which can ameliorate the impacts of extreme heat events as our exposure to such events continues to rise.

## Supporting information

S1 File Supplemental material – Regression analysis results. (DOCX)

S2 File Supplemental material – Sensitivity analyses. (DOCX)
