# Supplementary material for: Utilizing the Health Belief Model to understand heat mitigation behaviors in the United States: Results of an online panel survey
Source: PLoS One. 2025 Oct 14;20(10):e0334697. doi: 10.1371/journal.pone.0334697 (PMC12520335; doi:10.1371/journal.pone.0334697)
Supplement: S1 File — (DOCX) [file pone.0334697.s001.docx]

|  |  |  |  |  |  |
| --- | --- | --- | --- | --- | --- |
|  | Unadjusted Models | | | | |
|  | Estimate | Standard Error | p-value | Confidence Interval | R2 |
| Health Belief Model Constructs |  |  |  |  |  |
| Perceived Benefits | 0.6913 | 0.025 | <.0001 | (0.6422, 0.7404) | 0.311 |
| Perceived barriers | -0.1819 | 0.0138 | <.0001 | (-0.2089, -0.1548) | 0.056 |
| Perceived Susceptibility | 0.0697 | 0.00982 | <.0001 | (0.0504, 0.0889) | 0.0142 |
| Perceived Severity | 0.0707 | 0.014 | <.0001 | (0.0432, 0.0982) | 0.0081 |
| Self-Efficacy | 1.4898 | 0.0474 | <.0001 | (1.397, 1.5826) | 0.6035 |
| Cues to Action | 0.7959 | 0.0336 | <.0001 | (0.7302, 0.8617) | 0.3163 |
|  |  |  |  |  |  |
| Sex |  |  |  |  | 0.0023 |
| Female | 0.0825 | 0.0297 | 0.0055 | (0.0242, 0.1408) |  |
| Male (REF) | - | - | - | - |  |
| Political Affiliation |  |  |  |  | 0.0046 |
| Democrat | 0.1115 | 0.0398 | 0.0051 | (0.0335, 0.1895) |  |
| Independent | -0.1752 | 0.0444 | <.0001 | (-0.2624, -0.0881) |  |
| Republican (REF) | - | - | - | - |  |
| Age (in years) |  |  |  |  | 0.0258 |
| 18-29 | -0.3997 | 0.0576 | <.0001 | (-0.5127, -0.2867) |  |
| 30-39 | -0.1246 | 0.0586 | 0.0336 | (-0.2394, -0.00968) |  |
| 40-49 | -0.0458 | 0.0632 | 0.4689 | (-0.1696, 0.0781) |  |
| 50-64 | 0.1528 | 0.0552 | 0.0057 | (0.0446, 0.2611) |  |
| 65+ (REF) | - | - | - | - |  |
| Race/Ethnicity |  |  |  |  | 0.0078 |
| POC | -0.1565 | 0.0295 | <.0001 | (-0.2143, -0.0987) |  |
| NHW (REF) | - | - | - | - |  |
| Education |  |  |  |  | 0.0054 |
| < High school degree | -0.3353 | 0.1545 | 0.03 | (-0.6382, -0.0325) |  |
| High school degree/ GED | -0.074 | 0.0652 | 0.256 | (-0.2018, 0.0537) |  |
| Some college/2yr degree | 0.0314 | 0.0595 | 0.5975 | (-0.0853, 0.1481) |  |
| 4yr college degree | 0.0986 | 0.0633 | 0.1195 | (-0.0255, 0.2227) |  |
| Graduate degree (REF) | - | - | - | - |  |
| Income |  |  |  |  | 0.0132 |
| <$35,000 | -0.3018 | 0.0445 | <.0001 | (-0.3891, -0.2145) |  |
| $35,000-$54,999 | -0.00358 | 0.0519 | 0.9449 | (-0.1053, 0.0981) |  |
| $55,000-$99,999 | 0.0778 | 0.0468 | 0.0964 | (-0.0139, 0.1694) |  |
| $100,000+ (REF) | - | - | - | - |  |

Adjusted Model 1, Including all Health Belief Model Constructs

|  | Estimate | Standard Error | p-value | | Confidence Interval | R2 | AIC |
| --- | --- | --- | --- | --- | --- | --- | --- |
| Health Belief Model Constructs |  |  |  |  | | 0.6495 | 4412.128 |
| Perceived Benefits | 0.2668 | 0.0325 | <.0001 | (0.2032, 0.3304) | |  |  |
| Perceived barriers | 0.00652 | 0.0213 | 0.7599 | (-0.0353, 0.0483) | |  |  |
| Perceived Susceptibility | -0.00115 | 0.0158 | 0.9422 | (-0.0322, 0.0299) | |  |  |
| Perceived Severity | -0.0397 | 0.0245 | 0.1051 | (-0.0878, 0.00832) | |  |  |
| Self-Efficacy | 1.2916 | 0.0535 | <.0001 | (1.1868, 1.3964) | |  |  |
| Cues to Action | 0.4085 | 0.0388 | <.0001 | (0.3325, 0.4846) | |  |  |

| Adjusted Model 2, Including all Health Belief Model Constructs & Covariates | | | | |  |  |
| --- | --- | --- | --- | --- | --- | --- |
|  | Estimate | Standard Error | p-value | Confidence Interval | R2 | AIC |
| Health Belief Model Constructs |  |  |  |  | 0.6508 | 4412.105 |
| Perceived Benefits | 0.2633 | 0.0328 | <.0001 | (0.1989, 0.3276) |  |  |
| Perceived barriers | -0.00205 | 0.0222 | 0.9265 | (-0.0455, 0.0414) |  |  |
| Perceived Susceptibility | -0.00084 | 0.0163 | 0.9589 | (-0.0327, 0.0311) |  |  |
| Perceived Severity | -0.0422 | 0.0252 | 0.0937 | (-0.0915, 0.00714) |  |  |
| Self-Efficacy | 1.3044 | 0.0537 | <.0001 | (1.1991, 1.4097) |  |  |
| Cues to Action | 0.3948 | 0.0389 | <.0001 | (0.3186, 0.4711) |  |  |
|  |  |  |  |  |  |  |
| Sex |  |  |  |  |  |  |
| Female | 0.0685 | 0.0463 | 0.1393 | (-0.0223, 0.1594) |  |  |
| Male (REF) | - | - | - | - |  |  |
| Political Affiliation |  |  |  |  |  |  |
| Democrat | 0.0921 | 0.0623 | 0.1392 | (-0.03, 0.2143) |  |  |
| Independent | -0.0931 | 0.0677 | 0.1689 | (-0.2258, 0.0395) |  |  |
| Republican (REF) | - | - | - | - |  |  |
| Age (in years) |  |  |  |  |  |  |
| 18-29 | 0.029 | 0.0952 | 0.7606 | (-0.1577, 0.2157) |  |  |
| 30-39 | -0.0231 | 0.0866 | 0.7899 | (-0.1929, 0.1467) |  |  |
| 40-49 | 0.1328 | 0.095 | 0.162 | (-0.0534, 0.3191) |  |  |
| 50-64 | -0.1318 | 0.0797 | 0.0982 | (-0.288, 0.0244) |  |  |
| 65+ (REF) | - | - | - | - |  |  |
| Race/Ethnicity |  |  |  |  |  |  |
| POC | 0.0211 | 0.047 | 0.6541 | (-0.0711, 0.1133) |  |  |
| NHW (REF) | - | - | - | - |  |  |
| Education |  |  |  |  |  |  |
| < High school degree | -0.1447 | 0.2003 | 0.4701 | (-0.5373, 0.2479) |  |  |
| High school degree/ GED | 0.119 | 0.0942 | 0.2065 | (-0.0656, 0.3036) |  |  |
| Some college/2yr degree | 0.0146 | 0.0842 | 0.8621 | (-0.1504, 0.1797) |  |  |
| 4yr college degree | 0.0129 | 0.0901 | 0.8858 | (-0.1637, 0.1896) |  |  |
| Graduate degree (REF) | - | - | - | - |  |  |

| Adjusted Model 3, Including all Health Belief Model Constructs & Covariates, plus Income | | | | |  |  |
| --- | --- | --- | --- | --- | --- | --- |
|  | Estimate | Standard Error | p-value | Confidence Interval | R2 | AIC |
| Health Belief Model Constructs |  |  |  |  | 0.651 | 4415.934 |
| Perceived Benefits | 0.2641 | 0.0327 | <.0001 | (0.1999, 0.3282) |  |  |
| Perceived barriers | -0.0022 | 0.023 | 0.9238 | (-0.0472, 0.0428) |  |  |
| Perceived Susceptibility | -0.0015 | 0.0163 | 0.9263 | (-0.0334, 0.0304) |  |  |
| Perceived Severity | -0.042 | 0.0252 | 0.0962 | (-0.0914, 0.00748) |  |  |
| Self-Efficacy | 1.306 | 0.054 | <.0001 | (1.2002, 1.4118) |  |  |
| Cues to Action | 0.3955 | 0.0389 | <.0001 | (0.3193, 0.4718) |  |  |
|  |  |  |  |  |  |  |
| Sex |  |  |  |  |  |  |
| Female | 0.0697 | 0.0461 | 0.1308 | (-0.0207, 0.1602) |  |  |
| Male (REF) | - | - | - |  |  |  |
| Political Affiliation |  |  |  |  |  |  |
| Democrat | 0.092 | 0.0624 | 0.1402 | (-0.0303, 0.2143) |  |  |
| Independent | -0.0911 | 0.0677 | 0.1789 | (-0.2238, 0.0417) |  |  |
| Republican (REF) | - | - | - |  |  |  |
| Age (in years) |  |  |  |  |  |  |
| 18-29 | 0.0284 | 0.0959 | 0.7671 | (-0.1597, 0.2165) |  |  |
| 30-39 | -0.0225 | 0.0874 | 0.7969 | (-0.1939, 0.1489) |  |  |
| 40-49 | 0.1336 | 0.0951 | 0.1603 | (-0.0529, 0.32) |  |  |
| 50-64 | -0.1298 | 0.0798 | 0.1041 | (-0.2862, 0.0267) |  |  |
| 65+ (REF) | - | - | - |  |  |  |
| Race/Ethnicity |  |  |  |  |  |  |
| POC | 0.0204 | 0.0471 | 0.6648 | (-0.0719, 0.1126) |  |  |
| NHW (REF) | - | - | - |  |  |  |
| Education |  |  |  |  |  |  |
| < High school degree | -0.1231 | 0.2037 | 0.5456 | (-0.5225, 0.2762) |  |  |
| High school degree/ GED | 0.1114 | 0.0955 | 0.2434 | (-0.0758, 0.2985) |  |  |
| Some college/2yr degree | 0.000789 | 0.0849 | 0.9926 | (-0.1656, 0.1672) |  |  |
| 4yr college degree | 0.006 | 0.092 | 0.948 | (-0.1744, 0.1864) |  |  |
| Graduate degree (REF) | - | - | - |  |  |  |
| Income |  |  |  |  |  |  |
| <$35,000 | -0.0636 | 0.0761 | 0.4031 | (-0.2127, 0.0855) |  |  |
| $35,000-$54,999 | 0.0427 | 0.0816 | 0.6006 | (-0.1172, 0.2026) |  |  |
| $55,000-$99,999 | 0.0695 | 0.0693 | 0.3163 | (-0.0665, 0.2054) |  |  |
| $100,000+ (REF) | - | - | - | - |  |  |
