# Supplementary material for: Utilizing the Health Belief Model to understand heat mitigation behaviors in the United States: Results of an online panel survey
Source: PLoS One. 2025 Oct 14;20(10):e0334697. doi: 10.1371/journal.pone.0334697 (PMC12520335; doi:10.1371/journal.pone.0334697)
Supplement: S2 File — (DOCX) [file pone.0334697.s002.docx]

|  | Cut point 30  (25th percentile) | Cut point 34 (median) | Cut point 38  (75th percentile) |
| --- | --- | --- | --- |
|  | OR (95% CI) R2=0.52 | OR (95% CI) R2=0.65 | OR (95% CI) R2=0.52 |
| HBM Constructs |  |  |  |
| Perceived Benefits | 1.343 (1.246-1.449) | 1.301 (1.22-1.388) | 1.27 (1.171-1.378) |
| Perceived Barriers | 1.00 (0.949-1.053) | 0.998 (0.955-1.042) | 0.975 (0.936-1.016) |
| Perceived Susceptibility | 1.032 (0.991-1.075) | 0.999 (0.968-1.032) | 0.996 (0.966-1.026) |
| Perceived Severity | 0.957 (0.9-1.018) | 0.959 (0.913-1.007) | 0.948 (0.905-0.992) |
| Self-efficacy | 3.201 (2.874-3.565) | 3.686 (3.317-4.095) | 4.582 (3.76-5.584) |
| Cues to Action | 1.55 (1.429-1.681) | 1.484 (1.375-1.602) | 1.428 (1.298-1.572) |
| Race/Ethnicity |  |  |  |
| POC | 1.186 (0.96-1.466) | 1.043 (0.867-1.254) | 1.405 (1.156-1.709) |
| Non-Hispanic White (REF) | - | - | - |
| Sex |  |  |  |
| Female | 1.211 (0.984-1.49) | 1.147 (0.956-1.375) | 1.145 (0.944-1.388) |
| Male (REF) | - | - | - |
| Political Affiliation |  |  |  |
| Democrat | 0.99 (0.766-1.279) | 1.095 (0.886-1.355) | 0.633 (0.508-0.79) |
| Independent | 0.858 (0.659-1.118) | 0.91 (0.723-1.146) | 0.87 (0.679-1.113) |
| Republican (REF) | - | - | - |
| Age (in years) |  |  |  |
| 18-29 | 0.795 (0.557-1.135) | 1.037 (0.763-1.408) | 1.149 (0.848-1.558) |
| 30-39 | 0.932 (0.658-1.319) | 0.984 (0.74-1.309) | 1.228 (0.919-1.641) |
| 40-49 | 0.878 (0.613-1.258) | 1.15 (0.852-1.553) | 1.013 (0.736-1.393) |
| 50-64 | 0.841 (0.606-1.167) | 0.883 (0.676-1.152) | 1.044 (0.796-1.368) |
| 65+ (REF) | - | - | - |
| Education |  |  |  |
| No High School Degree | 0.687 (0.357-1.323) | 0.867 (0.509-1.475) | 0.74 (0.342-1.603) |
| High School/GED | 0.806 (0.569-1.143) | 1.128 (0.848-1.502) | 1.25 (0.929-1.682) |
| Some college/2yr degree | 0.772 (0.566-1.053) | 1.017 (0.782-1.321) | 1.093 (0.833-1.434) |
| 4 yr degree | 0.857 (0.619-1.185) | 1.015 (0.776-1.328) | 1.068 (0.807-1.412) |
| Graduate degree (REF) | - | - | - |
